# Driver Fatigue Detection Based on Convolutional Neural Networks Using EM-CNN

**DOI:** 10.1155/2020/7251280

**Published:** 2020-11-18

**Authors:** Zuopeng Zhao, Nana Zhou, Lan Zhang, Hualin Yan, Yi Xu, Zhongxin Zhang

**Affiliations:** ^1^School of Computer Science and Technology & Mine Digitization Engineering Research Center of Ministry of Education of the People's Republic of China, China University of Mining and Technology, Xuzhou 221116, China; ^2^School of Computer Science and Technology, China University of Mining and Technology, Xuzhou 221116, China

## Abstract

With a focus on fatigue driving detection research, a fully automated driver fatigue status detection algorithm using driving images is proposed. In the proposed algorithm, the multitask cascaded convolutional network (MTCNN) architecture is employed in face detection and feature point location, and the region of interest (ROI) is extracted using feature points. A convolutional neural network, named EM-CNN, is proposed to detect the states of the eyes and mouth from the ROI images. The percentage of eyelid closure over the pupil over time (PERCLOS) and mouth opening degree (POM) are two parameters used for fatigue detection. Experimental results demonstrate that the proposed EM-CNN can efficiently detect driver fatigue status using driving images. The proposed algorithm EM-CNN outperforms other CNN-based methods, i.e., AlexNet, VGG-16, GoogLeNet, and ResNet50, showing accuracy and sensitivity rates of 93.623% and 93.643%, respectively.

## 1. Introduction

A survey by the American Automobile Association's Traffic Safety Foundation found that 16–21% of traffic accidents were caused by driver fatigue [1]. According to Ammour et al., the probability of a traffic accident caused by driver fatigue is 46 times that of normal driving [2]. According to the “Special Survey and Investment Strategy Research Report of China's Traffic Accident Scene Investigation and Rescue Equipment Industry in 2019–2025,” there were 203,049 traffic accidents in China in 2017 (with a death toll of 63,372 and direct property loss of 1,21,131,300 yuan, the equivalent of 17,212,757.73 US dollars). To reduce the occurrence of such traffic accidents, it is of great practical value to study an efficient and reliable algorithm to detect driver fatigue.

There are currently four methods of driver fatigue detection:

1. Based on physiological indicators [3–6], Rohit et al. [7] analyzed the characteristics of electroencephalogram (EEG) using linear discriminant analysis and a support vector machine to detect driver fatigue in real time. However, most on-board physiological sensors are expensive and must be attached to human skin, which can cause driver discomfort and affect driver behavior.
2. Based on the driving state of the vehicle [8, 9], Ramesh et al. [10] used a sensor to detect the movement state of the steering wheel in real time to determine the degree of driver fatigue. However, the primary disadvantage of this method is that detection is highly dependent on the individual driving characteristics and the road environment. As a result, there is a high degree of randomness and contingency between the driving state of the vehicle and driver fatigue, which reduces the detection accuracy.
3. Based on machine vision [11, 12], Grace measured pupil size and position using infrared light of different wavelengths [13]. Yan et al. used machine vision to extract the geometric shape of the mouth shape [14]. An advantage of this method is that the facial features are noninvasive visual information that is unaffected by other external factors, i.e., driving state of the vehicle, individual driving characteristics, and road environment.
4. Based on information fusion, Wang Fei et al. combined physiological indicators and driving state of the vehicle to detect the driver fatigue state of the driver by collecting the EEG signal of the subject and the corresponding steering wheel manipulation data. However, the robustness of the test is affected by the individual's manipulation habits and the driving environment.

## 2. Related Work

To address the above difficulties, in this study, we consider deep convolutional neural networks (CNNs) [15–18]. CNNs have developed rapidly in the field of machine vision, especially for face detection [19, 20]. Viola and Jones [21] and Yang et al. [22] pioneered the use of the AdaBoost algorithm with Haar features to train different weak classifiers, cascading into strong classifiers for detecting faces and nonhuman faces. In 2014, Facebook proposed the DeepFace facial recognition system, which uses face alignment to fix facial features on certain pixels prior to network training and extracts features using a CNN. In 2015, Google proposed FaceNet, which uses the same face to have high cohesion in different poses, while different faces have low coupling properties. In FaceNet, the face is mapped to the feature vector of Euclidean space using a CNN and the ternary loss function [23]. In 2018, the Chinese Academy of Sciences and Baidu proposed PyramidBox, which is a context-assisted single-lens face detection algorithm for small, fuzzy, partially occluded faces. PyramidBox improved network performance by using semisupervised methods, low-level feature pyramids, and context-sensitive predictive structures [24]. CNN-based face detection performance is enhanced significantly by using powerful deep learning methods and end-to-end optimization. In this study, we combine eye and mouth characteristics and use a CNN rather than the traditional image processing method to realize feature extraction and state recognition [25, 26], and the necessary threshold is set to judge fatigue.

**Figure 1:**
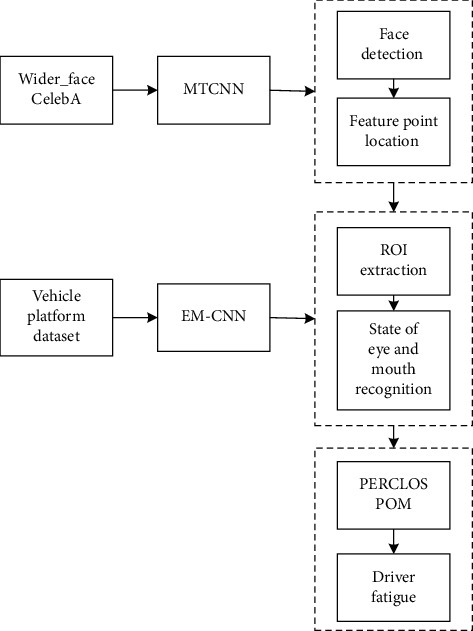
Flowchart of the proposed method to detect driver fatigue.

The proposed method to detect driver fatigue status comprises three components (Figure 1). First, the driver′s facial bounding box and the five feature points of the left and right eyes, nose, and the left and right corners of the mouth are obtained by an MTCNN [27]. Second, the states of the eyes and mouth are classified. Here, the region of interest (ROI) is extracted by the feature points, and the states of the eyes and mouth are identified by EM-CNN. Finally, we combine the percentage of eyelid closure over the pupil over time (PERCLOS) and mouth opening degree (POM) to identify the driver's fatigue status.

The primary contributions of this study are summarized as follows:

1. The EM-CNN, which is based on a state recognition network, is proposed to classify eye and mouth states (i.e., open or closed). In machine vision-based fatigue driving detection, blink frequency, and yawning are important indicators for judging driver fatigue. Therefore, this paper proposed a convolutional neural network that recognizes the state of the eyes and mouth to determine whether the eyes and mouths are open or closed. The EM-CNN can reduce the influence of factors such as changes in lighting, sitting, and occlusion of glasses to meet the adaptability to complex environments.
2. A method is developed to detect driver fatigue status. This method combines multiple levels of features by cascading two unique CNN structures. Face detection and feature point location are performed based on MTCNN, and the state of eyes and mouth is determined by EM-CNN.
3. Binocular images (rather than monocular images) are detected to obtain abundant eye features. For a driver's multipose face area, detecting only monocular′s information can easily cause misjudgment. To obtain richer facial information, a fatigue driving recognition method based on the combination of binocular and mouth facial features is proposed, which utilizes the complementary advantages of various features to improve the recognition accuracy.

## 3. Proposed Methodology

### 3.1. Face Detection and Feature Point Location

**Figure 2:**
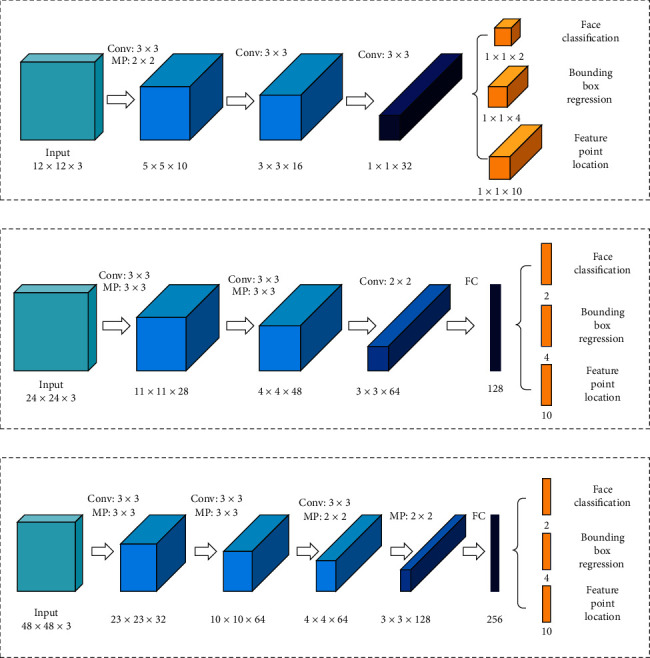
MTCNN architecture: (a) P-Net, (b) R-Net, and (c) O-Net.

Face detection is challenging in real-world scenarios due to changes in driver posture and unconstrained environmental factors, such as illumination and occlusion. By using the depth-cascading multitasking MTCNN framework, face detection and alignment can be completed simultaneously, the internal relationship between the two is exploited to improve the performance, and the global face features are extracted; thus, the positions of the face, left and right eyes, nose, and the left and right corners of the mouth can be obtained. The structure of the MTCNN is shown in Figure 2. The MTCNN comprises three cascaded subnetworks, i.e., P-Net (proposal network), R-Net (refined network), and O-Net (output network), which are detected face and feature point position from coarse to fine.

P-Net: first, an image pyramid is constructed to obtain images of different sizes. These images are then input to the P-Net in sequence. A fully convolutional network is employed to determine whether a face is included in a 12 × 12 area at each position, thereby obtaining a bounding box of the candidate face area and its regression vector. Then, the candidate face window is calibrated with the frame regression vector, and nonpolar large value suppression is employed to remove highly overlapping candidate face regions [28, 29].

R-Net: the candidate face area obtained by P-Net input, and the image size is adjusted 24 × 24. The candidate face window is screened by bounding box regression and nonmaximum value suppression. In comparison with the P-Net, the network structure adds a connection layer to obtain a more accurate face position.

O-Net: similar to the R-Net, in the O-Net, the image size is adjusted to 48 × 48, and the candidate face window is screened to obtain the final face position and five feature points.

**Figure 3:**
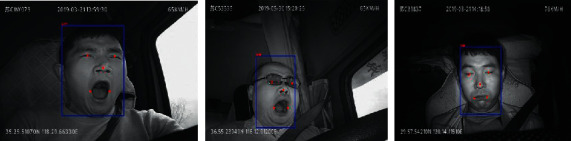
Face detection and feature point location using the MTCNN.

The MTCNN performs face detection via a three-layer cascade network that performs face classification, bounding box regression, and feature point location simultaneously. It demonstrates good robustness and is suitable for real driving environments. The result is shown in Figure 3.

### 3.2. State of the Eye and Mouth Recognition

#### 3.2.1. ROI Extraction

Generally, most eye detection methods only extract one eye to identify a fatigue state. However, when the driver's head shifts, using information from only a single eye can easily cause misjudgment. Therefore, to obtain more eye information and accurately recognize the eye state, the proposed method extracts a two-eye image to determine whether the eyes are open or closed.

The position of the driver's left and right eyes is obtained using the MTCNN network. Here, the position of the left eye is a1 (*x*1, *y*1), the position of the right eye is a2 (*x*2, *y*2), the distance between the left and right eye is *d*1, and the width of the eye image is *w*1. The height is *h*1, according to the proportion of the face “three courts and five eyes,” the binocular images are intercepted, and the correspondence between width and height is expressed as follows:

(1) $$\begin{array}{c} d1=\sqrt{x1-x2^{2}+y1-y2^{2}},\\ w1=1.5d1,\\ h1=d1. \end{array}$$

A driver's mouth region changes significantly when talking and yawning. Here, the positions of the left and right corners of the mouth are obtained using the MTCNN network. The position of the left corner of the mouth is b1 (*x*3, *y*3), and the position of the right corner of the mouth is *b*2(*x*4, *y*4). The distance between the left and right corners is *d*2, the width of the mouth image is *w*2, and the height is *h*2, similar to the eye region extraction. The correspondence between width and height is expressed as follows:

(2) $$\begin{array}{c} d2=\sqrt{x3-x4^{2}+y3-y4^{2}},\\ w2=d2,\\ h2=d2. \end{array}$$

#### 3.2.2. EM-CNN Architecture

**Figure 4:**
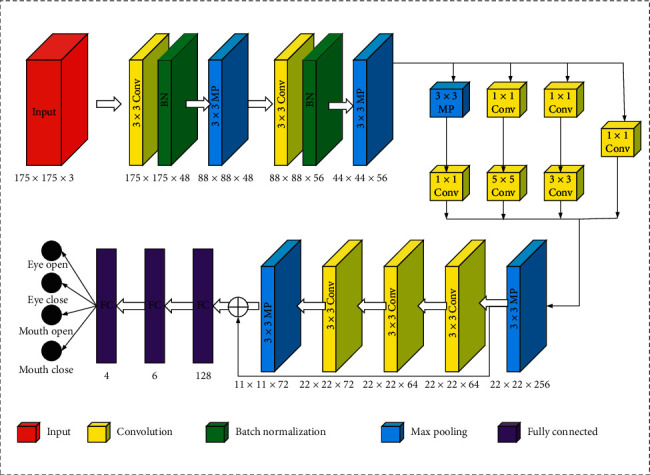
EM-CNN structure.

After extracting the eyes and mouth regions, it is necessary to evaluate the state of the eyes and mouth to determine whether they are open or closed. The proposed method employs EM-CNN for eye and mouth state recognition. The network structure is shown in Figure 4.

In a real driving environment, the acquired images of the driver's eyes and mouth are different in size; thus, the size of the input image is adjusted to 175 × 175, and a feature map of 44 × 44 × 56 is obtained by two convolution pools. Here, the size of the convolution kernel in the convolutional layer is 3 × 3, and the step size is 1. The size of the convolution kernel in the pooled layer is 3 × 3, and the step size is 2. To avoid reducing the size of the output image and causing partial information loss at the edges of the image, a layer of pixels is filled along the edge of the image before the convolution operation. Then, the 1 × 1, 3 × 3, 5 × 5 convolution layers and 3 × 3 pooling layer are used to increase the adaptability of the network to the size. The feature map of 44 × 44 × 256 is obtained through another pooling. Then, after passing through a residual block, there are three layers of convolution in the residual block, and the layer is pooled, and an 11 × 11 × 72 feature map is output. The feature map is then converted to a one-dimensional vector in the fully connected layer, and the number of parameters is reduced by random inactivation to prevent network overfitting. Finally, the classification result (i.e., eyes are open or closed, and the mouth is open or closed) is output by softmax.

### 3.3. Fatigue State Detection

When the driver enters the fatigue state, there is usually a series of physiological reactions, such as yawning and closing the eyes. According to the EM-CNN, multiple states of the eyes and mouth are acquired, and the fatigue state of the driver is evaluated by calculating the eye closure degree PERCLOS and mouth opening degree POM.

#### 3.3.1. PERCLOS

The PERCLOS parameter indicates the percentage of eye closure time per unit time [30]:

(3) $$\begin{array}{c} \text{PERCLOS}=\frac{\sum_{i}^{N}f_{i}}{N}\times 100\%. \end{array}$$

Here,*f*_*i*_ represents the closed frame of the eye, ∑_*i*_^*N*^*f*_*i*_ represents the number of closed-eye frames per unit time, and *N* is the total number of frames per unit time. To determine the fatigue threshold, 13 video frame sequences were collected to test and calculate its PERCLOS value. The result showed that when PERCLOS is greater than 0.25, the driver is in the closed-eye state for a long time, which can be used as an indicator of fatigue.

#### 3.3.2. POM

Similar to PERCLOS, POM represents the percentage of mouth closure time per unit time:

(4) $$\begin{array}{c} \text{POM}=\frac{\sum_{i}^{N}f_{i}}{N}\times 100\%. \end{array}$$

Here,*f*_*i*_ indicates the frame with the mouth open, ∑_*i*_^*N*^*f*_*i*_ indicates the number of open mouth frames per unit time, and *N* is the total number of frames per unit time. When POM is greater than 0.5, it can be judged that the driver is in the opened mouth state for a long time, which can also be used as an indicator of fatigue. Greater values for these two indicators suggest higher degrees of fatigue.

#### 3.3.3. Fatigue State Recognition

After the neural network pretraining is completed, the fatigue state is identified based on the fatigue threshold of PERCLOS and POM. First, the face and feature point positions of the driver frame image are obtained by the MTCNN, and the ROI area of the eyes and mouth is extracted. Then, the state of the eyes and mouth is evaluated by the proposed EM-CNN. Here, the eye closure degree and mouth opening degree of the continuous frame image are calculated, and the driver is determined to be in a fatigue state when the threshold is reached.

## 4. Experimental Results

### 4.1. Dataset Description

**Figure 5:**
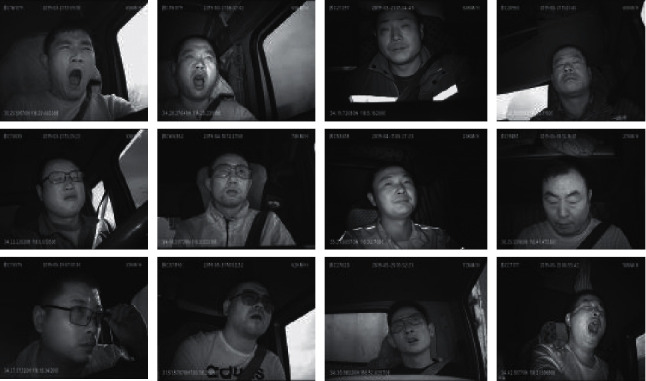
Sample images from the driver dataset.

**Figure 6:**
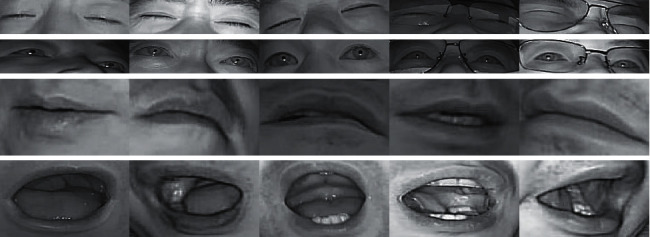
Examples of training sets.

The driver driving images used in this study were provided by an information technology company called Biteda. A total of 4000 images of a real driving environment were collected (Figure 5). This paper runs a holdout for the experimental results and divides the datasets directly into a 3/7 ratio. The datasets were divided into four categories, i.e., open eyes (2226 images; 1558 training and 668 test samples), closed eyes (1774 images; 1242 training and 532 test samples), open mouth (1996 images; 1397 training and 599 test samples), and closed mouth (2004 images; 1,403 training and 601 test samples). Examples of training sets are shown in Figure 6. The test sets are similar to the training sets, but with different drivers.

### 4.2. Implementation Details

**Figure 7:**
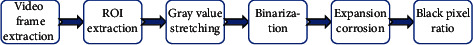
Calculation of eye closure based on machine vision.

**Figure 8:**
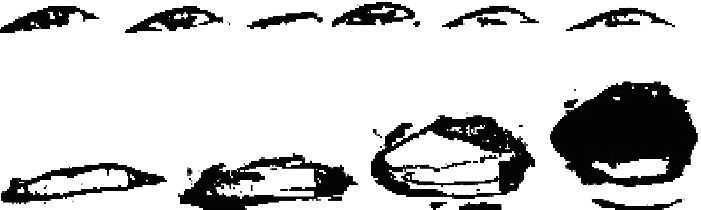
(a) Images showing changes in the blinking process. (b) Images showing changes in the yawning process.

During the driving process, the amplitude of the mouth change under normal conditions is less than that in the fatigue state, which is easy to define. In contrast, the state change of the eye while blinking is difficult to define; thus, the eye state is defined by calculating eye closure based on machine vision. A flowchart of this method is shown in Figure 7. To define the state of the eyes, the ROI is binarized, and the binary image is smoothed using expansion and erosion techniques. The area of the binocular image can be represented by a black area, and the number of black pixels and the total number of pixels of the ROI are counted.  Figure 8 shows the processed eyes and mouth. Here, the ratio of the two is calculated as the eye closure degree, and the eye state is evaluated using a threshold value of 0.15. When the ratio is greater than 0.15, the eye is in the open state, and when the ratio is less than 0.15, the eye is closed.

During training, the batch size is set to 32, and the optimization method is Adam (with a learning rate of 0.001). An epoch means that all training sets are trained once, and 100 epochs are trained using the training set.

The training and testing of MTCNN and EM-CNN are based on Python 3.5.2 and Keras 2.2.4 with Tensorflow 1.12.0 as the back end. A computer with an Nvidia GTX 1080 Ti GPU, 64-bit Windows 7, and 4 GB of memory was used as the experimental hardware platform.

### 4.3. Performance of EM-CNN

**Figure 9:**
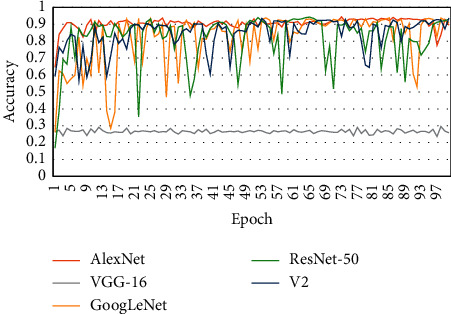
Comparison of accuracy of various networks.

**Figure 10:**
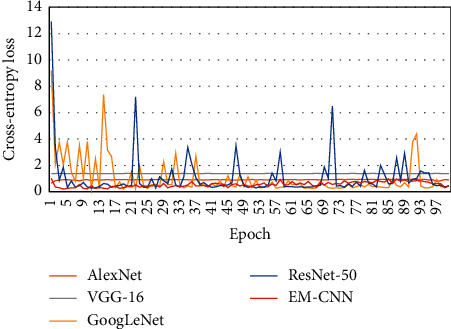
Comparison of loss of various networks.

To verify the efficiency of EM-CNN, we implemented several other CNN methods, i.e., AlexNet [16], VGG-16 [31], GoogLeNet [32], and ResNet50 [33]. The accuracy, sensitivity, and specificity results are shown in Table 1. The proposed EM-CNN showed an accuracy of 93.623%, sensitivity of 93.643%, and specificity of 60.882%. The proposed EM-CNN outperformed the compared networks.  Figure 9 shows a comparison of the accuracy obtained by different networks, and a comparison of cross-entropy loss for these networks is shown in Figure 10.

**Figure 11:**
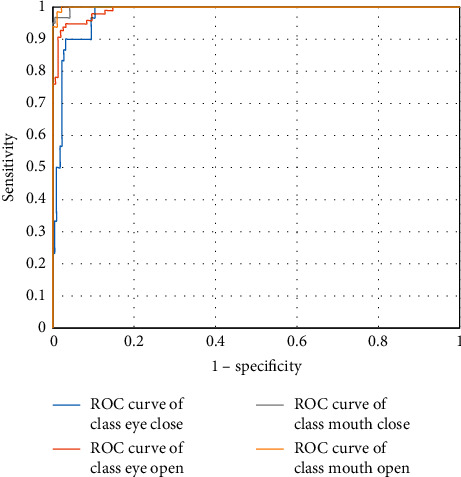
ROC curves for four classifications.

Figure 11 shows that the proposed EM-CNN is more sensitive and specific to the state of the mouth than the eyes because drivers show large changes to the mouth and less interference in the fatigue state.  Table 2 gives four classifications under the receiver operating characteristic (ROC) curve. The larger the area under the curve (AUC) value, the better the classification effect. The experimental results demonstrate that the classification effect of the proposed EM-CNN on the mouth state is better than that for the eyes.

### 4.4. Fatigue State Recognition

**Figure 12:**
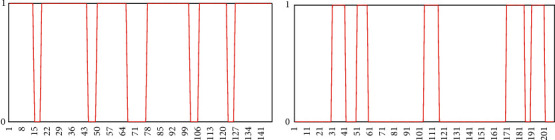
Comparison of driver's eye state changes under (a) normal and (b) fatigue conditions.

Combined with the temporal correlation of eye state changes, judging by the blink frequency, fatigue driving is to mark the state of the eyes in the video frame sequence. If the detected eye state is open, it is marked as “1”; otherwise, it is marked as “0,” and the blink process in the video frame sequence can be expressed as a sequence of “0” and “1.” Figure 12 reflects changes in the open and closed states of the eyes.

**Figure 13:**
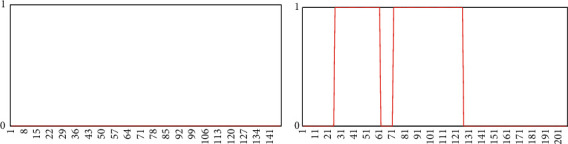
Comparison of driver's mouth state changes under (a) normal and (b) fatigue conditions.

The frequency of yawning is used to judge the fatigue driving and is to mark the mouth state in the video frame sequence. If the detected mouth state is open, it is marked as “1,” otherwise it is marked as “0”, then the beat in the video frame sequence, the yawning process can be expressed as a sequence of “0” and “1.” Figure 13 reflects the changes in the mouth state of the driver under normal and fatigue conditions.

**Figure 14:**
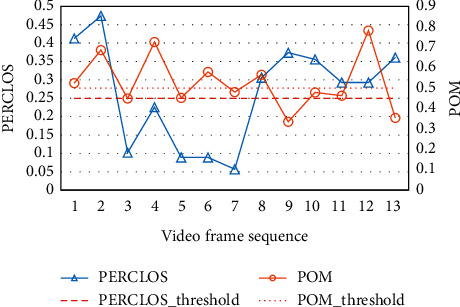
Threshold of PERCLOS and POM.

Thresholds are related to different methods used in different studies, so the existing results cannot be used directly. Thresholds for PERCLOS and POM should be obtained from experiments. Thirteen video frame sequences of drivers in a real driving environment were collected and experimented with the converted video stream to frame image for recognition, PERCLOS and POM values were calculated. The results are shown in Figure 14. The fatigue state can be identified according to the fatigue threshold. The test results show that when PERCLOS reaches 0.25, it can be judged that the driver is in the closed-eye state for a long time. When POM reaches 0.5, the driver is in the open mouth state for a long time. Note that the degree of fatigue is greater as these indicators take greater values.

## 5. Conclusions

The method of detecting driver fatigue based on cascaded MTCNN and EM-CNN is expected to play an important role in preventing car accidents caused by driving fatigue. For face detection and feature point location, we use the MTCNN architecture of a hierarchical convolutional network, which outputs the facial bounding box and the five feature points of the left and right eyes, nose, and the left and right mouth corners. Then, the ROI of the driver image is extracted using the feature points. To evaluate the state of the eyes and mouth, this paper has proposed the EM-CNN-based detection method. In an experimental evaluation, the proposed method demonstrated high accuracy and robustness to real driving environments. Finally, driver fatigue is evaluated according to PERCLOS and POM. The experimental results demonstrate that when PERCLOS reaches 0.25 and POM reaches 0.5, the driver can be considered in a fatigue state. In the future, it will be necessary to further test the actual performance and robustness of the proposed method. In addition, we will implement the method on a hardware device.

## Figures and Tables

**Table 1:** Performance comparison of different networks.

Network	Accuracy (%)	Sensitivity (%)	Specificity (%)
AlexNet	89.565	86.065	58.724
VGG-16	25.797	50	50
GoogLeNet	91.015	89.906	60.412
ResNet-50	92.899	92.292	58.547
EM-CNN	93.623	93.643	60.882

**Table 2:** AUC for four classifications.

State	AUC (%)
Eye close	97.913
Eye open	99.1
Mouth close	99.854
Mouth open	99.918

## Data Availability

The data used to support the findings of this study are available from the corresponding author upon request.
